# IgA subclasses have different effector functions associated with distinct glycosylation profiles

**DOI:** 10.1038/s41467-019-13992-8

**Published:** 2020-01-08

**Authors:** Ulrike Steffen, Carolien A. Koeleman, Maria V. Sokolova, Holger Bang, Arnd Kleyer, Jürgen Rech, Harald Unterweger, Martin Schicht, Fabian Garreis, Jonas Hahn, Fabian T. Andes, Fabian Hartmann, Madelaine Hahn, Aparna Mahajan, Friedrich Paulsen, Markus Hoffmann, Günter Lochnit, Luis E. Muñoz, Manfred Wuhrer, David Falck, Martin Herrmann, Georg Schett

**Affiliations:** 1 Department of Internal Medicine 3—Rheumatology and Immunology, Friedrich-Alexander-University Erlangen-Nürnberg (FAU) and Universitätsklinikum Erlangen, Erlangen, Germany; 2 Leiden University Medical Center, Center for Proteomics and Metabolomics, Glycomics and Clinical Proteomics Group, Leiden, Netherlands; 3 Orgentec Diagnostika, Mainz, Germany; 4 Section of Experimental Oncology and Nanomedicine (SEON), ENT Department, Friedrich-Alexander-University Erlangen-Nürnberg (FAU) and Universitätsklinikum Erlangen, Erlangen, Germany; 5 Institute of Functional and Clinical Anatomy, Friedrich Alexander University Erlangen-Nürnberg, Erlangen, Germany; 6 Protein Analytics, Institute of Biochemistry, Faculty of Medicine, Justus-Liebig-University Giessen, Giessen, Germany

**Keywords:** Antibodies, Translational immunology, Rheumatology

## Abstract

Monomeric serum immunoglobulin A (IgA) can contribute to the development of various autoimmune diseases, but the regulation of serum IgA effector functions is not well defined. Here, we show that the two IgA subclasses (IgA1 and IgA2) differ in their effect on immune cells due to distinct binding and signaling properties. Whereas IgA2 acts pro-inflammatory on neutrophils and macrophages, IgA1 does not have pronounced effects. Moreover, IgA1 and IgA2 have different glycosylation profiles, with IgA1 possessing more sialic acid than IgA2. Removal of sialic acid increases the pro-inflammatory capacity of IgA1, making it comparable to IgA2. Of note, disease-specific autoantibodies in patients with rheumatoid arthritis display a shift toward the pro-inflammatory IgA2 subclass, which is associated with higher disease activity. Taken together, these data demonstrate that IgA effector functions depend on subclass and glycosylation, and that disturbances in subclass balance are associated with autoimmune disease.

## Abstract (web summary)

Immunoglobulin A (IgA) has two subclasses, IgA1 and IgA2, but differential effects on inflammation are unclear. Here the authors show that IgA2, when compared with IgA1, has stronger pro-inflammatory functions associated with changed glycosylation and higher disease scores in patients with rheumatoid arthritis.

## Introduction

Immunoglobulin A (IgA) is the most frequently produced antibody in the human body^1^. The majority of IgA is secreted as a dimer into mucosal tissues, where it has an important function in intestinal microbiota homeostasis^2^. However, in humans, IgA also represents the second most abundant immunoglobulin in the serum, reaching concentrations of 1–3 mg/ml^1^. In the past few years, it has become clear that serum IgA has particular immunological functions independent from the role of secretory IgA in the gut. Of note, serum IgA strongly differs from secretory IgA. Whereas secretory IgA is dimeric, serum IgA is composed mainly of monomers. The monomeric form and the lack of the secretory component enable serum IgA to bind to Fcα-receptor I (FcαRI) expressed by myeloid cells, such as monocytes, neutrophils, and some subsets of macrophages and dendritic cells (DCs)^3^. Serum IgA can thereby induce pro-inflammatory responses, such as the release of cytokines and chemokines, phagocytosis, degranulation, and formation of neutrophil extracellular traps (NETs)^4^. Opsonization of gut bacteria with IgA even converts anti-inflammatory intestinal CD103^+^ DCs to a pro-inflammatory phenotype, which protects against invading pathogens, but might also result in chronic inflammation^5^. Evidence exists that serum IgA contributes to autoimmune diseases, such as inflammatory bowel disease^6,7^, autoimmune skin blistering diseases^8,9^, or rheumatoid arthritis (RA)^10–12^ as well as to transplant rejection^13^. In addition, IgA has gained interest as a therapeutic antibody against cancer cells, as it activates neutrophil-mediated antibody-dependent cellular cytotoxicity better than IgG^14,15^.

By contrast, several anti-inflammatory effects of serum IgA have also been described, proposing a protective role of IgA against autoimmunity and autoinflammation. Crosslinking of FcαRI or stimulation with monomeric IgA inhibited the production of pro-inflammatory cytokines and induced IL-10 expression in human monocytes and monocyte-derived DCs^16,17^. In addition, injection of human serum IgA reduced paw swelling in FcαRI transgenic mice with collagen-induced arthritis or collagen antibody-induced arthritis^18^.

The observed discrepancies between pro-inflammatory and anti-inflammatory effects of serum IgA demonstrate that more knowledge is required to understand the mechanisms of IgA-mediated effector functions. FcαRI initiates activating but also inhibitory signals depending on the intensity of its activation^19^. However, how binding of IgA to FcαRI is regulated and modulated remains unclear. FcαRI belongs to the same family as Fcγ-receptors (FcγR)^20^ that are important for IgG effector functions. For IgG, it is well established that subclass-specific structural differences and Fc glycosylation affect affinity to FcγR^21,22^; similar regulatory mechanisms might exist for IgA.

Humans possess two IgA subclasses, IgA1 and IgA2, that differ mainly in the structure of their hinge region and in the number of glycosylation sites^3^. In serum, IgA1 is predominant against IgA2 with a ratio of 9:1, whereas in mucosal tissues, IgA1 and IgA2 are more evenly distributed. Of note, with the exception of chimpanzees, gorillas, and gibbons, humans are the only species possessing two IgA subclasses^23^. By contrast, mice express only one IgA isotype and do not have a functional homologue to FcαRI^3^. The fact that mice have a completely different IgA system that can’t be compared to humans might explain why subclass specific effects of IgA have scarcely been examined to date. However, further knowledge about the effects of IgA subclass and glycosylation is needed to understand the two-sided function of serum IgA as inducer of both tolerance and inflammation.

In this study, we explore differences in the effector functions of human serum IgA1 and IgA2 on myeloid cells. We show that IgA2 induces NET formation and cytokine production by neutrophils and macrophages to a substantially higher degree than IgA1. Mass spectrometry analysis of *O*-glycans and *N*-glycans indicates that IgA1 and IgA2 are differentially glycosylated, and IgA1 effector function increases after enzymatic removal of sialic acid or whole *N*-glycans. In addition, differences in the IgA1:IgA2 ratio amongst autoantibodies from patients with RA are associated with disease severity. In summary, our study identifies the IgA subclass as a major regulator of IgA effector function, which might have broad implications for the development of human autoimmune diseases.

## Results

### IgA2 has stronger pro-inflammatory effects than IgA1

**Fig. 1:**
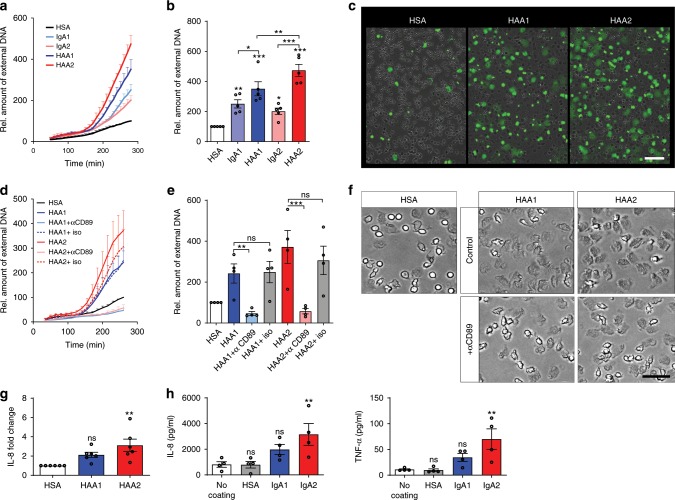
IgA2 activates neutrophils and macrophages more potently than IgA1. **a**–**c** Human neutrophils were stimulated with 200 µg/ml of monomeric or heat aggregated (=HAA) IgA1 and IgA2, or with human serum albumin (HSA). Neutrophil extracellular trap (NET) formation was evaluated by staining extracellular DNA with Sytox Green. **a** NET formation over time. **b** Relative amount of extracellular DNA at 280 min after stimulation normalized on HSA treatment; *n* = 5 donors. **c** Representative images at 280 min after stimulation. Scale bar = 50 µm. **d**–**f** NET formation of human neutrophils with 200 µg/ml HAA1 or HAA2 in the presence of 10 µg/ml blocking antibody against FcαRI (=αCD89) or isotype control (=iso). **d** NET formation over time. **e** Relative amount of extracellular DNA at 260 min after stimulation normalized on HSA treatment; *n* = 4 donors. **f** Representative images at 35 min after stimulation. Scale bar = 20 µm. **g** IL-8 released by neutrophils 4 h after stimulation with HSA, HAA1, or HAA2; *n* = 6 donors. **h** Cytokines released by macrophages 6 h after seeding in wells coated with HSA, IgA1, or IgA2; *n* = 4 donors. Significances were tested with paired one-way ANOVA followed by Bonferroni correction for selected pairs of columns **b**, **d** or Dunnet’s correction **g**, **h**. *p < 0,05; **p < 0,01; and ***p < 0,001. Data are presented as mean ± s.e.m. **a**, **d** or scatter blots with bars showing mean ± s.e.m. **b**, **e**, **g**, **h**. Source data are provided as a Source Data file.

To investigate putative differences in the effector functions of the two IgA subclasses, we isolated IgA from pooled serum of healthy donors and separated it into IgA1 and IgA2. IgA containing immune complexes and particles coated with IgA have been reported to be potent inducers of NET formation^24,25^. We thus first analyzed the effects of IgA1 and IgA2 in a human NET formation assay. We isolated neutrophils from the blood of healthy donors and stimulated them with 200 µg/ml of serum IgA1 and IgA2 that was either monomeric or had been heat aggregated to mimic immune complexes. As a control for the protein load, human serum albumin (HSA) was used. Neutrophils challenged with IgA displayed an increased NET formation rate compared to cells treated with HSA (Fig. 1a–c). This effect was more pronounced with heat aggregated IgA (=HAA) than with monomeric IgA. While we did not see a significant difference between monomeric IgA1 and IgA2, heat aggregated IgA2-induced NET formation more potently than heat aggregated IgA1. This difference was consistent among various concentrations of IgA (Supplementary Fig. 1a, b).

A blocking antibody against FcαRI (=CD89) completely abrogated heat aggregated IgA-induced NET formation (Fig. 1d, e), while no effect was seen with the isotype control. Of note, most neutrophils rapidly became adherent in the presence of aggregated IgA (Fig. 1f), but not if FcαRI was blocked. Together, these data confirm that activation of neutrophils by aggregated IgA is FcαRI dependent, as it has been published before^24,25^. As expected, blocking of FcγRI (=CD64) or FcγRIII (=CD16) did not affect NET formation (Supplementary Fig. 1c, d).

In addition to the increased NET formation, neutrophil stimulation with heat aggregated IgA2 resulted in an increased release of IL-8, while heat aggregated IgA1 had no verifiable effect compared to the HSA-treated control (Fig. 1g). To exclude major alterations in the IgA complex formation to be a reason for the observed differences in neutrophil stimulation, we measured the complex size of heat aggregated IgA1 and IgA2 with dynamic light scattering. Complexes of both IgA subclasses showed equal sizes with a mean diameter of ~28 nm (Supplementary Fig. 1e, f). Electron microscopy images confirmed the IgA complexes to be rather small in size (Supplementary Fig. 1g). In addition, all IgA samples had been verified to be free of endotoxin to avoid endotoxin-mediated side effects.

NET formation can be induced via different pathways^26^. To better characterize IgA-mediated NET formation, we used the inhibitors diphenylene iodonium (DPI) and GSK484 directed against neutrophil NADPH oxidase (NOX) and peptidylarginine deiminase 4 (PAD4), respectively. Phorbol 12-myristate 13-acetate (PMA) was used as positive control for NOX-dependent NET formation and ionophore A32187 served as positive control for PAD4-dependent NET formation. Heat aggregated IgA1 and IgA2-induced NET formation was completely blocked in the presence of DPI (Supplementary Fig. 2), indicating the formation of reactive oxygen species by NOX to be the main pathway. In contrast, PAD4 activation does not seem to play a major role as the inhibitor GSK484 showed no effect.

To determine if the observed stronger activation with IgA2 is cell-type-specific or a global phenomenon, we next investigated the effects of IgA1 and IgA2 on human monocyte-derived macrophages. To mimic the presence of immune complexes without the need of heat aggregation, we immobilized IgA1, IgA2, or the protein load control HSA on the surface of cell culture plate wells. Differentiated macrophages were seeded into these wells and after 6 h the supernatant was analyzed for the presence of cytokines. Macrophages stimulated with immobilized IgA2 secreted substantially increased amounts of IL-8 and tumor necrosis factor α (TNF-α), although the levels of TNF-α were overall relatively low (Fig. 1h). Immobilized IgA1 did not significantly increase the secretion of these cytokines.

### IgA1 and IgA2 induce different signaling in neutrophils

To elucidate why the two IgA subclasses exert different effects on myeloid cells, we investigated signaling pathways involved in IgA-mediated NET formation. As described above, IgA complexes drive NET formation via FcαRI. Similar to FcγRs, the activation of FcαRI leads to immunoreceptor tyrosine-based activation motif phosphorylation in the common Fc receptor gamma chain with subsequent recruitment and activation of spleen tyrosine kinase (Syk). Downstream, several pathways are activated, including Ca^2+^ release, formation of phosphatidylinositol (3,4,5)-trisphosphate and activation of the mitogen-activated protein kinase/extracellular signal-regulated kinase (MAPK/Erk) pathway^27^.

**Fig. 2:**
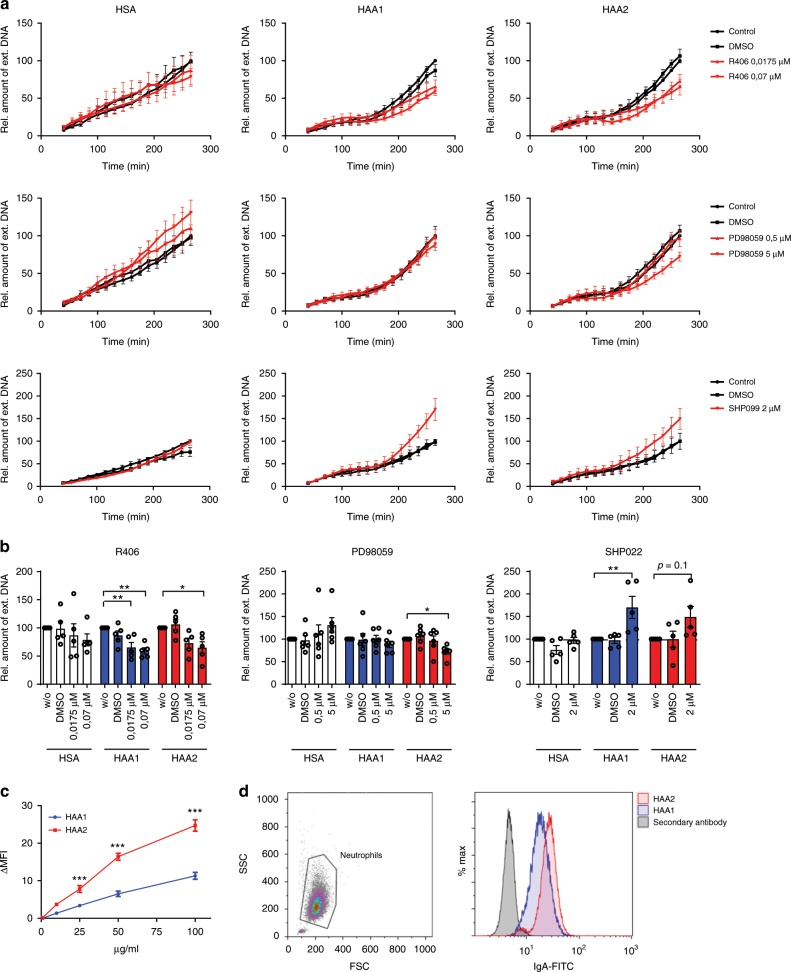
IgA2 induces stronger binding and activating signaling to neutrophils than IgA1. **a**, **b** Human neutrophils were stimulated with 100 µg/ml of heat aggregated IgA1 and IgA2 (=HAA1 and HAA2) or with human serum albumin (=HSA) in the presence of inhibitors for Syk (=R406), Erk (=PD98059) or Shp-2 (=SHP099) at the indicated concentrations. Neutrophil extracellular trap (NET) formation was evaluated by staining extracellular DNA with Sytox Green. **a** NET formation over time and **b** relative amount of extracellular DNA at 265 min after stimulation normalized on treatment without inhibitors; *n* = 5–6 donors. **c** Binding of HAA1 and HAA2 to neutrophils measured by flow cytometry; *n* = 4 donors. **d** Representative blots of flow cytometry analysis showing neutrophil gating and fluorescence intensity. FSC, forward scatter; SSC, side scatter. Significances were tested with paired one-way ANOVA followed by Dunnett’s correction **b** or Bonferroni correction for selected pairs of columns **c**. *p < 0,05; **p < 0,01; and ***p < 0,001. Data are presented as mean ± s.e.m. **a**, **c** or scatter blots with bars showing mean ± s.e.m. **b**. Source data are provided as a Source Data file.

To get a first hint if both IgA subclasses activate FcαRI to a similar degree, we isolated human neutrophils and stimulated them with 100 µg/ml of heat aggregated IgA1 and IgA2, or HSA in the presence of the Syk inhibitor R406 or the Erk inhibitor PD98059. As expected, inhibition of Syk reduced NET formation to ~60–70% of the values reached with heat aggregated IgA1 or IgA2 without inhibitor (Fig. 2a, b). Interestingly, inhibition of Erk only diminished NET formation triggered by IgA2 complexes, but had no effect on IgA1 complex-mediated NET formation. The basal NET formation rate in the presence of the protein load control HSA was not influenced by the two inhibitors, demonstrating that the observed inhibitory effects were specific for IgA-triggered NET formation. In addition, the solvent dimethyl sulfoxide (DMSO) had no effect on NET formation. Cytotoxic effects of the inhibitors could be excluded (Supplementary Fig. 3a, b), while the solvent DMSO displayed a minor cytotoxic effect by itself.

Activating signals of immunoreceptors can be blocked by simultaneous inhibitory signals that lead to the activation of phosphatases, like Src homology 2 (SH2) domain containing inositol phosphatase, SH2-containing tyrosine phosphatase-1 (Shp-1) or Shp-2 via immunoreceptor tyrosine-based inhibitory motif phosphorylation^28^. Inhibition of the phosphatase Shp-2 with the inhibitor SHP099 markedly increased aggregated IgA1-induced NET formation (Fig. 2a, b). We also saw a tendency toward increased NET formation in the presence of Shp-2 inhibitor, when neutrophils were stimulated with aggregated IgA2. However, this effect was less pronounced and not significant. Basal NET formation in the presence of HSA was not affected by the Shp-2 inhibitor. Together these data suggest that IgA2 complexes initiate a robust neutrophil activation via activation of Syk and Erk, while IgA1 complex-mediated signaling seems to be less stable and at least partially blocked by inhibitory Shp-2 signals.

To test if the observed differences in signaling could arise from a different binding affinity of the IgA subclasses, we measured the relative amount of aggregated IgA1 and IgA2 bound to neutrophils by flow cytometry, using a secondary antibody that recognizes both IgA subclasses. We observed a consistently higher binding of IgA2 complexes to neutrophils compared to IgA1 complexes (Fig. 2c, d). Of note, this effect was not due to a higher affinity of the secondary antibody to IgA2, as polystyrene beads coated with IgA2 even displayed lower fluorescence signals than IgA1-coated polystyrene beads (Supplementary Fig. 3c).

### IgA1 and IgA2 have different glycosylation patterns

**Fig. 3:**
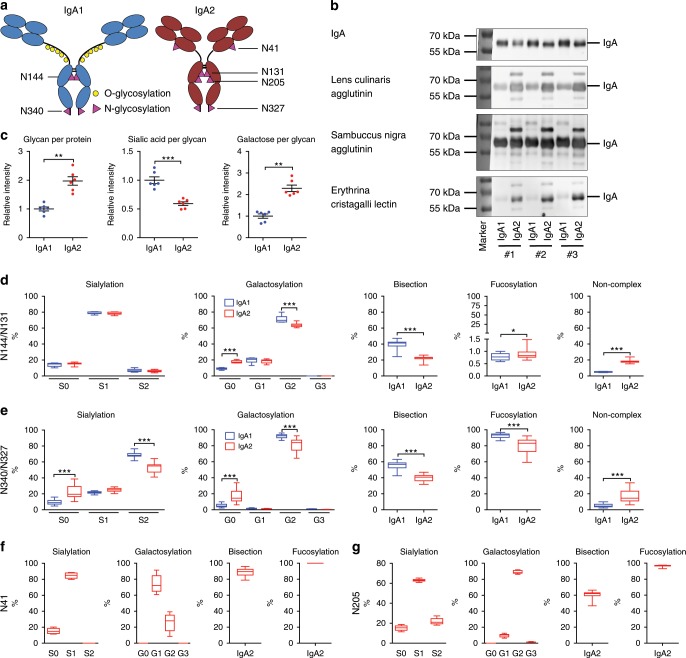
IgA1 and IgA2 are differentially glycosylated. **a** Schematic overview of glycosylation sites in IgA1 and IgA2. Bold letters indicate glycosylation sites present in both IgA subclasses. **b** Representative lectin blots of IgA1 and IgA2 isolated from sera of healthy donors using antibodies against IgA or lectins against the core structure of *N*-glycans (=lens culinaris agglutinin), terminal α2,6-linked sialic acid (=sambuccus nigra agglutinin), and terminal galactose (=erythrina christagalli lectin). **c** Quantification of the lectin blots; *n* = 6 donors. **d**–**f** Mass spectrometric quantification of sialyation, galactosylation, bisection, fucosylation, and the presence of noncomplex structures for the glycosylation sites N144/N131 **d**, N340/N327 **e**, N41 **f**, and N205 **g**; *n* = 12 donors. Significances were tested with paired two-sided Student’s *t*-test **c**–**g** or paired one-way ANOVA followed by Bonferroni correction for selected pairs of columns **d**–**g**. *p < 0,05; **p < 0,01; and ***p < 0,001. Data are presented as scatter plots with mean ± s.e.m. **c** or box plots with medians and inter-quartile ranges + whiskers ranging from min to max **d**–**g**. Source data are provided as a Source Data file.

For IgG, it has been well established, that the binding affinity to FcγR and subsequent signaling is strongly regulated by Fc glycosylation^29^. IgG glycans are in general biantennary complex-type oligosaccharides containing a highly conserved heptamer core of mannose and *N*-acetylglucosamine residues. To this core, additional sugar residues can be attached, such as galactose, sialic acid, bisecting *N*-acetylglucosamine, and fucose (Supplementary Fig. 4a). For IgG, it has been shown that the composition of these sugars greatly affects IgG effector functions^29^. Mouse and in vitro experiments indicate that for example the presence of terminal galactose or sialic acid plays an important role in the decision if IgG acts pro- or anti-inflammatory^30–32^. While IgG contains only one conserved *N*-glycosylation site, IgA1 holds two and IgA2 even four conserved sites for *N*-glycosylation^33^ (Fig. 3a). In addition, IgA1 possesses several *O*-glycosylation sites in its hinge region. We thus hypothesized that differences in the glycan composition, such as the presence of terminal sialic acid, might affect IgA effector functions as well.

To test if differences in sialylation might contribute to the different effector functions of the IgA subclasses, we performed lectin blot analysis on isolated serum IgA1 and IgA2 from healthy donors. As expected, we detected two times more *N*-glycans (detected with lens culinaris lectin) in IgA2 compared to IgA1 (Fig. 3b, c), which fits to the reported number of four *N*-glycosylation sites in IgA2 versus only two *N*-glycosylation sites in IgA1^33^. When normalized on the signal for the *N*-glycan, the signal for terminal α2,6-linked sialic acid (detected with sambuccus nigra agglutinin) was reduced by half in IgA2 compared to IgA1. In contrast, the signal for free galactose (detected with erythrina christagalli lectin) was doubled in IgA2 compared to IgA1.

To determine the compositions of individual *N*-glycans, we performed mass spectrometry analysis of glycopeptides from isolated serum IgA1 and IgA2 from 12 healthy donors (age 40–62 years; 75% female; Fig. 3d–g). Remarkably, we observed only very little variation between the donors. With respect to the glycosylation sites N144/N131 and N340/N327 that are present in both subclasses, IgA2 displayed in general less galactosylation, less sialylation (only at N340/N327), less bisecting *N*-acetylglucosamine, and less fucosylation (only at N340/N327) of complex type glycans. In addition, IgA2 showed relatively high levels of noncomplex glycans (=high mannose or hybrid type; see Supplementary Fig. 4b, c). The two *N*-glycosylation sites unique to IgA2 were mainly monosialylated and had a high level of bisection and fucosylation. Of note, for the glycosylation site N340/N327, we could detect in both IgA subclasses a second—truncated—glycopeptide form in which the C-terminal tyrosine was missing. This phenomenon has been described before^34^, but it is so far unclear if it is of biological relevance. Compared to the nontruncated version, the truncated glycopeptides displayed quite similar glycan compositions, with overall slightly increased sialylation, galactosylation, and fucosylation levels (Supplementary Fig. 5a). Noncomplex type glycans were not observed for the truncated version.

In addition to *N*-gylcans, we investigated the *O*-glycosylation sites of IgA1. We detected on average 4.5 *O*-glycans per heavy chain with an overall galactosylation of 80% and sialylation of 50% (Supplementary Fig. 5b).

Together, our mass spectrometry data revealed a consistently lower sialylation in IgA2 compared to IgA1 with on average 25% less sialic acid per *N-*glycan and also per IgA molecule when counting *N*- and *O*-glycans together (Supplementary Fig. 5c).

In addition to conserved Fc glycosylation, antibodies can introduce further glycosylation sites within the variable domains of the Fab region caused by somatic hypermutation^35^. To date, the function of Fab glycosylation is unclear, but it could influence antigen binding, effector function or the half-life of immunoglobulins. To test for the presence of Fab glycosylation in serum IgA, we performed polyacrylamide gel electrophoresis with two independent preparations of IgA1 and IgA2 together with IgG from sera of healthy donors, followed by lectin blot analysis with lens culinaris agglutinin to detect *N*-glycans (Supplementary Fig. 5d, e). As IgA2 contains a conserved glycosylation site (N41) in the Fab region of the heavy chain, we concentrated on the light chain. As expected, we found a signal for *N*-glycosylation in the light chains of IgG. In contrast, no signal could be observed in IgA1 or IgA2, suggesting that IgA does not or only to very little extent contain variable domain Fab glycosylation.

### IgA1 effector functions are controlled by Fc glycosylation

**Fig. 4:**
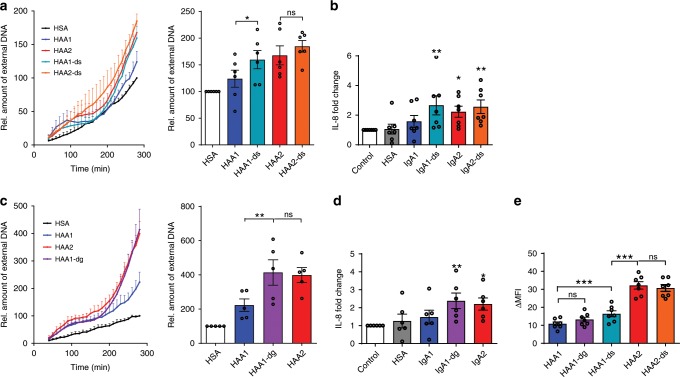
Removal of sialic acid or all *N*-glycans increases pro-inflammatory activity of IgA1. **a** Human neutrophils were stimulated with 100 µg/ml of human serum albumin (=HSA) and native or desialylated (=-ds) heat aggregated IgA1 and IgA2 (=HAA1 and HAA2). NET formation was evaluated by staining extracellular DNA with Sytox Green. Shown is NET formation over time and the relative amount of extracellular DNA at 280 min after stimulation normalized on HSA treatment; *n* = 6 donors. **b** IL-8 released by macrophages 6 h after seeding in wells coated with HSA, IgA1, desialylated IgA1 (=IgA1-ds), IgA2 or desialylated IgA2 (=IgA2-ds); *n* = 7 donors. **c** Human neutrophils were stimulated with 100 µg/ml of human serum albumin (=HSA) and native or deglycosylated (=-dg) heat aggregated IgA1 and IgA2 (=HAA1 and HAA2); *n* = 5 donors. **d** IL-8 released by macrophages 6 h after seeding in wells coated with HSA, IgA1, deglycosylated IgA1 (=IgA1-dg) or IgA2; *n* = 6 donors. **e** Binding of heat aggregated IgA1 and IgA2 (=HAA1 and HAA2) that has been desialylated (=-ds) or deglycosylated (=-dg) on neutrophils measured by flow cytometry; *n* = 7 donors. Significances were tested with paired one-way ANOVA followed by Bonferroni correction for selected pairs of columns **a**, **c**, **e** or Dunnet’s correction **b**, **d**. *p < 0,05; **p < 0,01; and ***p < 0,001. Data are presented as mean ± s.e.m. **a**, **c** or scatter blots with bars showing mean ± s.e.m. **a**–**e**. Source data are provided as a Source Data file.

To investigate if the higher levels of sialic acid in IgA1 glycans might be responsible for its lack of inflammatory effector functions, we removed sialic acid of serum IgA1 and IgA2 by treatment with the enzyme neuraminidase (Supplementary Fig. 6a). Indeed, desialylation of IgA1 increased its capacity to induce NET formation by neutrophils (Fig. 4a) and IL-8 production by macrophages (Fig. 4b) at levels comparable to IgA2. By contrast, the activity of IgA2 was not noticeably affected by neuraminidase treatment. Interestingly, the complete removal of *N*-glycans with PNGase F had the same effect on IgA1 (Fig. 4c, d) standing in strong contrast to experiences made with IgG, which is unable to exert most effector functions if its Fc glycan is missing^36^. The effects of deglycosylation on IgA2 could not be investigated, as we were not able to remove all sugars from IgA2 with PNGase F (Supplementary Fig. 6b).

Dynamic light scattering measurements showed a size increase in heat aggregated IgA after desialylation and deglycosylation (Supplementary Fig. 7a), which was equal for IgA1 and IgA2. In addition, flow cytometry analysis revealed a slightly increased binding of aggregated desialylated IgA1 compared to aggregated native IgA1 to neutrophils, while binding of aggregated deglycosylated IgA1 was not altered (Fig. 4e). However, binding of aggregated IgA2 was still much stronger. Together, these data indicate that IgA effector functions are not only determined by binding affinity, but that other factors are involved as well. Again, we tested if binding of the secondary antibody was affected by desialylation or deglycosylation using polystyrene beads, but we could not see a difference between beads coated with native, desialylated, or deglycosylated IgA1 (Supplementary Fig. 7b).

### Increased IgA2:IgA1 ratios correlate with RA disease scores

**Fig. 5:**
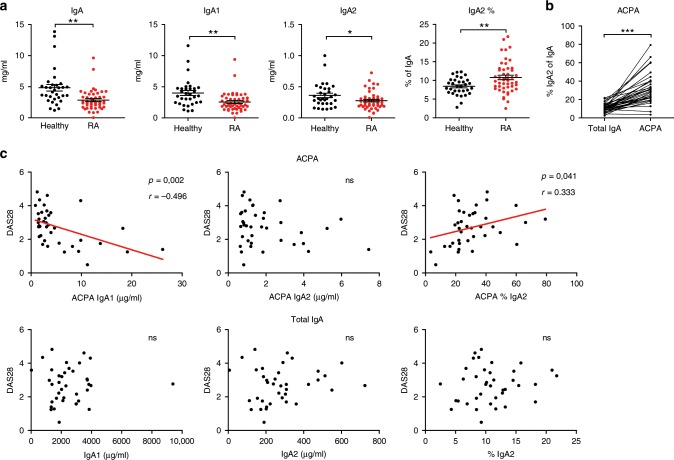
A high IgA2 percentage in ACPA correlates with higher RA disease activity. **a** Amount of serum IgA, IgA1, and IgA2 as well as IgA2 percentage in patients with RA (*n* = 48) and healthy controls (*n* = 32). **b** IgA2 percentage of total IgA compared to ACPA in the serum of ACPA-positive RA patients (*n* = 38). **c** Correlation of the IgA1 and IgA2 amount, and IgA2 percentage in total serum IgA and IgA-ACPA with the disease activity score (DAS) 28 score of ACPA positive RA patients (*n* = 38). Significances were tested with two-sided Student’s *t*-test using Welch’s correction **a**, paired two-sided Student’s *t*-test **b**, or Pearson **c**. *p < 0,05; **p < 0,01; and ***p < 0,001. Data are presented as scatter plots with mean ± s.e.m. Source data are provided as a Source Data file.

The presence of autoantibodies from the IgA class is associated with a worse disease prognosis, increased synovitis and joint destruction in patients with RA^11,12,37^. Neutrophils and macrophages play a major role in the manifestation of synovitis^38^. As we found IgA2 to activate these cells, we hypothesized that especially autoantibodies of the IgA2 subclass might contribute to disease severity in RA. To investigate if the IgA levels or subclass distribution are altered in patients with RA, we measured the amount of total IgA, IgA1, and IgA2 in the serum of 48 patients with RA and compared them to the levels of 32 age and sex-matched healthy controls (see Supplementary Table 1 for characteristics of patients and controls). RA patients displayed slightly reduced levels of serum IgA, IgA1, and IgA2 (Fig. 5a). As expected, in both groups IgA1 levels were about ten times higher than IgA2 levels. However, the IgA subclass ratio was slightly changed in RA patients leading to a higher percentage of the pro-inflammatory IgA2. Interestingly, investigating a larger cohort of healthy subjects, we found markedly increased serum IgA levels in men compared to women (Supplementary Fig. 8a). This difference was based on higher IgA1 levels in men, while IgA2 levels were the same in both groups. Regarding subclass distribution, women thus displayed a higher IgA2 percentage in IgA. To determine if the observed difference in the IgA1:IgA2 ratio between healthy subjects and RA patients is sex dependent, we reanalyzed our data separately for men and women. However, we found the same tendencies for both sexes (Supplementary Fig. 8b), although the increase in IgA2 percentage was smaller in women with RA, most likely due to the higher basal value in healthy women.

We next investigated the IgA subclass distribution in autoantibodies against citrullinated proteins (ACPA) that are highly specific for RA. Interestingly, compared to total serum IgA, the IgA1:IgA2 ratio in ACPA was strongly shifted toward IgA2 (Fig. 5b). We further tested whether the levels of IgA ACPA and their subclass distribution correlated with disease activity in RA using a validated instrument (disease activity score 28; DAS28). Interestingly, higher levels of IgA1 ACPA correlated with lower DAS28 scores (Fig. 5c), suggesting a protective effect of IgA1 autoantibodies. Conversely, a high percentage of IgA2 in ACPA correlated with higher disease scores. This effect was specific for ACPA as the levels and proportion of total serum IgA1 and IgA2 showed no correlation. Together, these data are in line with our findings that IgA2, but not IgA1 acts pro-inflammatory on myeloid cells and indicate that IgA2 autoantibodies might contribute to disease manifestation in patients with RA.

## Discussion

Immunoglobulins are an essential component of the adaptive immune system to fight pathogens. In human serum, IgG is the most frequent immunoglobulin class, but, with concentrations between 1–3 mg/ml, IgA is also abundantly present in human serum^1^. However, the biological relevance of IgA and the regulation of its effector functions are still insufficiently characterized. In the past few years, increasing evidence suggests that IgA is not only involved in gut homeostasis, but also plays an important role in the regulation of immune responses. In addition, several autoimmune diseases are associated with the presence of autoantibodies of the IgA class^4^. However, the role of IgA is controversially discussed and pro- as well as anti-inflammatory effects have been described^27^.

The present study shows that the two IgA subclasses IgA1 and IgA2 act differently on neutrophils and macrophages. While we did not detect pronounced effector functions of IgA1, IgA2 promoted inflammatory reactions like enhanced NET formation and the expression of inflammatory cytokines. This different behavior of IgA1 and IgA2 could be an explanation for the at first sight controversial effects of IgA observed in previous studies. Many studies describing anti-inflammatory effects of IgA worked with total serum IgA^16–19,39^, which is to 90% composed of IgA1 that did not induce inflammatory cytokine production in our hands. On the other hand, the described associations with autoimmune diseases are most likely due to specific autoantibodies that may have an increased IgA2 fraction, as we have observed for RA-specific ACPA. In contrast to our findings, Hansen et al. reported similar pro-inflammatory effects of IgA1 and IgA2 on CD103^+^ DC^5^. However, they performed their experiments in the presence of the toll-like receptor 2 ligand Pam3CSK4. It is likely that the presence of secondary inflammatory stimuli, which we did not use in our study, generally increases pro-inflammatory signaling of IgA, as it is known for IgG^40,41^ and thereby overcomes differences between the two IgA subclasses.

For both IgA subclasses, the induction of NET formation was diminished by the Syk inhibitor R406. This was expected as FcαRI, like FcγR, signals via the common Fcγ receptor chain with subsequent activation of Syk^3^. Interestingly, we found that IgA2, but not IgA1-mediated NET formation was additionally affected by Erk inhibition, indicating Erk activation by IgA2 complexes. Erk is reported to be a downstream target of FcαRI^27^. Whether activation of Erk by IgA2 complexes is due to a more persistent binding of IgA2 than IgA1 complexes to FcαRI (quantitative difference) or due to an additional activation of coreceptors (qualitative difference) remains to be determined in future studies. We found that IgA2 complexes elicit a stronger binding to neutrophils than IgA1 complexes. However, from this experiment we can’t conclude if the increased binding is due to an increased affinity to FcαRI or to other, yet unknown receptors.

According to the UniProt-structures P01876 and P01877, the constant regions of IgA1 and IgA2 share 88% amino acid sequence identity^42,43^. Apart from the hinge region, most differences are changes of single amino acids. However, so far it has not been investigated if these differences alter the affinity to FcαRI or to other receptors. In addition, detailed analysis of subclass specific IgA glycosylation, especially of the shared glycosylation sites N144/N131 and N340/N327 are scarce as most studies measured the glycosylation of these sites only for IgA1^34,44^. We found one study of Chandler et al. investigating all glycans of serum IgA1 and IgA2^45^. However, they had a very low sample size and a high variation, which might explain why they did not see glycosylation differences between the IgA subclasses.

As described in former studies, we found that IgA contained high amounts of galactose, sialic acid, bisecting *N*-acetylglucosamine, and fucose (with exception of N144/N131, that was mainly unfucosylated)^34,44,45^. We found highly significant differences between IgA1 and IgA2 glycosylation. Overall, IgA1 was more sialylated and galactosylated, and displayed higher levels of bisecting *N*-acetylglucosamine compared to IgA2 on the shared glycosylation sites. Interestingly, we also found glycans that were not of complex, but of high-mannose or hybrid type. This phenomenon stands in strong contrast to IgG, for which glycans of noncomplex type have been found only in very little amounts^46,47^. In addition, IgG is in general much less sialylated and galactosylated than IgA^48^. This fact could be explained by different glycan positions in IgA and IgG with IgA glycans being more exposed to the outside of the immunoglobulin molecule^33^, which might result in a greater accessibility and thus higher galactosylation and sialylation rate.

It is currently not known how the distinct glycosylation sites of IgA affect its effector functions. There are some studies reporting that glycans are not involved in binding of IgA to FcαRI^49,50^, but also one study showing an increased association rate of deglycosylated IgA to FcαRI^51^. Our data clearly show that enzymatic removal of terminal sialic acid or the whole glycan increases the pro-inflammatory capacities of IgA1, demonstrating that IgA glycosylation is important for IgA effector function. It has to be noted that desialylation and deglycosylation led to an increased IgA complex size after heat aggregation. However, we do not believe that this size increase was responsible for the stronger pro-inflammatory effects observed after sugar removal, as we saw the same increase in both IgA subclasses and desialylation did not alter the effector function of IgA2 despite the increased complex size. Furthermore, we observed only a mild increase in binding of IgA1 complexes to neutrophils after desialylation and no change after deglycosylation, indicating that the binding affinity to FcαRI might indeed not be dramatically influenced by the glycan composition. However, the glycan composition might affect binding to other receptors and thereby influence effector functions as it has been described for IgG^52^. In mice, secretory IgA has been found to bind to specific ICAM-3 grabbing nonintegrin-related 1 (SIGNR1), which is an important mediator of anti-inflammatory effects of sialylated IgG^53^. It is possible that IgA1, but not IgA2 binds to similar receptors via its sialylated glycans. Loss of these glycans or of sialic acid would abolish the interaction of IgA1 to these receptors and lead to increased pro-inflammatory effects, as we have seen in our experiments. However, at the moment, these concepts remains speculative and need to be investigated in future studies.

Of note, in addition to *N*-glycans, IgA1 possesses several sites for *O*-glycosylation in its hinge region that accounts for half of the sialic acid residues present in the IgA1 molecule. *O-*glycan-bound sialic acid most likely contributed to the high difference in sialylation detected in our lectin blot analysis between IgA1 and IgA2, and might also contribute to the differences in IgA1 and IgA2 effector functions. Low levels in *O*-glycan galactosylation are associated with IgA immune complex formation and mesangial IgA deposition in IgA-mediated nephropathy^54–56^. If *O*-glycan sialylation plays a role as well is less understood, but might be possible. From our experiments, we can’t say if the increased pro-inflammatory effects of desialylated IgA1 are because of *N*- or *O*-glycan desialylation. However, treatment with PNGaseF, cleaving only *N*-glycans, also profoundly increased the inflammatory properties of IgA1, demonstrating the importance of *N*-glycoslyation for the regulation of IgA1 effector functions. Whether both *N*-glycosylation sites are involved in this regulation and whether *O*-glycans play a role as well needs to be clarified in further studies.

Interestingly, we found that in RA-specific ACPA, compared to total serum IgA, the IgA1:IgA2 ratio was shifted toward pro-inflammatory IgA2. This shift could explain published data, showing that ACPA of the IgA class are associated with poor disease prognosis, higher inflammation, and more severe joint destruction in RA patients^10–12,37^. Unfortunately, these studies have not discriminated between IgA1 and IgA2. We found that an increased IgA2 fraction in IgA-ACPA, but not in total IgA correlated with higher disease scores in RA patients, indicating IgA2 ACPA to be involved in disease progression. The exact mechanisms still have to be defined, but it is likely that IgA2 immune complexes enhance the invasion of immune cells into inflamed tissues by stimulating neutrophils and macrophages to release IL-8, a potent attractor of neutrophils^57^. Van der Steen et al. showed that neutrophils migrate to IgA deposits in a skin blistering disease model^8^ supporting this hypothesis. Of note, the presence of IgA1 ACPA correlated with lower disease scores which fits to the described protective effect of serum IgA in a murine inflammatory arthritis model^18^ given that serum IgA is mainly composed of IgA1.

Together, our data propose a concept how serum IgA effector functions are regulated in humans. IgA1, which makes the majority of serum IgA, seems to be important for immune homeostasis, while IgA2 exerts inflammatory effects. In addition, our data demonstrate that IgA glycosylation controls IgA effector functions. Further research is needed to elucidate how exactly IgA glycosylation influences the inflammatory capacity of IgA. In addition, the relevance of autoantibody glycosylation for autoimmune disease development in general has still to be proven by therapeutic manipulation in patients. However, our findings provide an important step for a better understanding of the potential contribution of IgA to autoimmune diseases and for the generation of therapeutic IgA.

## Methods

### IgA isolation and generation of IgA complexes

IgA was isolated from pooled sera of healthy donors using peptide M agarose (Invivogen) according to the manufacturer’s instructions. After an additional washing step with 0.1 M glycine buffer (pH 5.0) to remove nonspecifically bound proteins, bound IgA was eluted with 0.1 M glycine buffer (pH 2.7), immediately neutralized using 1 M Tris buffer (pH 9), and dialyzed against phosphate-buffered saline (PBS) (pH 7.2). IgA1 and IgA2 were separated using jacalin agarose (Thermo Scientific) according to the manufacturer’s instructions. IgA2 was collected with the flow through. IgA1 bound to the agarose and was eluted with 0.1 M galactose buffer. Both IgA fractions were concentrated and buffered to PBS using Amicon Ultra Centrifugal Filters (Merck) followed by treatment with Triton-X114 (Sigma) to remove possible traces of endotoxins. Traces of Triton-X114 were subsequently removed with detergent removal columns (Thermo Scientific). Protein concentration was determined with the DC protein assay (Bio-Rad). Purity of IgA1 and IgA2 was tested with western blot analysis using antibodies against IgA1 and IgA2 (Southern biotech), as well as Coomassie staining to exclude contaminations by other serum proteins.

IgA aggregates were obtained by heat aggregation at 63 °C for 30 min.

### Isolation and stimulation of neutrophils

Neutrophils were freshly isolated from blood with ethylenediaminetetraacetic acid (EDTA) as coagulant from healthy donors by standard density gradient centrifugation using Ficoll (Lymphoflot, BioRad). After taking the polymorph nuclear cell (PMN) fraction, erythrocytes were hypotonically lysed in two cycles with sterile water. All stimulation assays were performed in RPMI medium without phenol red and supplemented with 1% penicillin/streptomycin and 1% glutamin (both Gibco, Invitrogen).

For NET formation assays, neutrophils were plated in 96-well microplates (Ibidi) at a concentration of 0.75 × 10^6^ cells/ml together with 2.5 µM Sytox Green (Invitrogen) for visualization of extracellular DNA. After addition of the indicated amounts of IgA, heat aggregated IgA or HSA (Sigma), the plate was placed in a live cell imaging chamber of a BZ-X710 microscope (Keyence) and kept at 37 °C and 5% CO_2_. Every 10–15 min pictures were taken. The area of Sytox Green-positive events was analyzed by Photoshop CS5 software and normalized on the number of cells present at the first measured time point.

For Fc receptor studies, blocking antibodies against FcαRI (clone MIP8a, BioRad), FcγRI (clone 10.1), FcγRIII (clone 3G8), or isotype control (mouse IgG1, κ, all Biolegend) were used at a concentration of 10 µg/ml. Neutrophils were incubated with the antibodies for 5 min before aggregated IgAs were added.

For the characterization of pathways involved in NET formation, inhibitors against NOX (=DPI) and PAD4 (=GSK484, all Sigma) were used at a concentration of 1 µM. In addition, the stimulators PMA and ionophore A23187 (all Sigma) were used at concentrations of 10 nM and 4 µM, respectively.

For signaling studies inhibitors against Syk (=R406), Erk (=PD98059) and Shp-2 (=SHP099, all Axon Medchem) were used at the indicated concentrations. Neutrophils were incubated with the inhibitors or DMSO as solvent control for 5 min before aggregated IgAs or HSA were added.

For measurement of cytokine production, neutrophils were seeded at a density of 10 × 10^6^ cells/ml and stimulated with 200 µg/ml of heat aggregated IgA or HSA. After 4 h, the supernatant was centrifuged for 5 min at 4 °C and 10,000 × *g* to remove cell debris and stored at −20 °C for further analysis. Concentrations of cytokines and chemokines were measured by multiplex bead technology (Legendplex; BioLegend) and quantified by cytofluorometry with a Gallios cytofluorometer and subsequent analysis with Kaluza Analysis 2.1 software (both Beckman Coulter).

### Investigation of cell viability

PMNs were isolated as described above, resuspended in RPMI medium without phenol red that was supplemented with 1% penicillin/streptomycin, 1% glutamin (both Gibco, Invitrogen) and 100 µg/ml HSA (Sigma), and seeded in a 96-well cell culture plate (200 µl with 150,000 PMNs per well). PMNs were first incubated with the indicated inhibitors or DMSO for 30 min at 37 °C and 5% CO_2_. In addition, some PMNs were heated for 5 min to 65 °C to induce cell death (=positive control). A volume of 20 µl of alamarBlue reagent (Thermo scientific) was added to each well. Viability of the cells was analyzed in an Infinite® 200 PRO plate reader (Tecan) at 37 °C and 5% CO_2_ by the assessment of the absorbance at the wavelengths 570 and 595 nm every hour for a total of 4 h.

### Generation and stimulation of macrophages

Human monocytes were purified by plastic adhesion of peripheral blood mononuclear cells that had been isolated from EDTA-blood of healthy donors using a Ficoll gradient (Lymphoflot, BioRad). Macrophages were generated in α-Mem (Invitrogen) supplemented with 10% fetal bovine serum (Biochrome) and 1% penicillin/streptomycin (Invitrogen) in the presence of 30 ng/ml macrophage colony-stimulating factor (Peprotech). After 6 days, macrophages were detached using Cell Stripper (Corning) and seeded at a concentration of 0.5 × 10^6^ cells/ml on plates coated with 50 µg/ml IgA1, IgA2, or HSA. After 4 h, the supernatant was centrifuged for 5 min at 4 °C and 10,000 × *g* to remove cell debris and stored at −20 °C for further analysis. Cytokine concentrations were measured as described above.

### Measurement of IgA complex size

IgA complexes were generated by incubation of IgA1 or IgA2 with a concentration of 5 mg/ml at 63 °C for 30 min. The hydrodynamic size of the aggregates dispersed in PBS was acquired by dynamic light scattering with a Malvern Nano ZS (Malvern Panalytical) in backscattered mode (173°) and at 25 °C.

### Transmission electron microscopy

IgA complexes were generated by incubation of IgA1 or IgA2 with a concentration of 535 mg/ml at 63 °C for 30 min. For the transmission electron microscopy (TEM) negative staining, aggregated IgA solutions were diluted in buffer containing 10 mM Hepes and 140 mM NaCl_2_ to a final concentration of 15 µg/ml and coated on a 200 mesh copper grid supported carbon film (Formovar, Plano). After 2 min incubation, the grid was washed with one droplet of H_2_O and air-dried for 30 min at room temperature. Staining was performed with 1% uranyl acetate for 2 min. The staining solution was removed with filter paper and the grid was washed again with one droplet of H_2_O and air-dried for 30 min at room temperature.

The visualization of IgA aggregates was performed with a transmission electron microscope (TEM 109, Zeiss) operating at 80 kV and magnifications between 50,000× and 140,000×.

### Gel electrophoresis and lectin blotting

IgA1 and IgA2 isolated from sera of healthy donors was resolved on a 10% sodium dodecyl sulfate (SDS)–polyacrylamide gel under reducing conditions and transferred to a polyvinylidene difluoride membrane (Merk). After blocking with 3% deglycosylated gelatin (Sigma), blots were incubated with horseradish peroxidase (HRP)-labeled goat anti-human IgA (1:10,000; #2050-05; Southern Biotech), biotinylated lens culinaris agglutinin (5 µg/ml; #B-1045) for the detection of the core glycan, biotinylated erythrina cristagalli lectin (5 µg/ml; #B-1145) for galactose detection, or biotinylated sambuccus nigra lectin (2 µg/ml; #B-1305; all vector laboratories) for sialic acid detection, followed by incubation with HRP-labeled streptavidin (1:500; # DY998; R&D). Detection was performed with chemoluminescence reagent (ECL; Thermo Scientific) on a chemilumineszenz-imager (Celvin S 320+, Biostep). Band intensities were quantified with Photoshop CS5 software.

For protein staining of the light chains, IgA1, IgA2, and IgG isolated from mixed sera of healthy donors was resolved on a 15% SDS–polyacrylamide gel under reducing conditions with subsequent staining with Imperial™ Protein Stain (Thermo Scientific) according to the manufacturers’ instructions.

Uncropped blot images can be found in the Source Data file.

### IgA glycosylation analysis by mass spectrometry

An equivalent of 20 µg of IgA1 or IgA2 eluate isolated from sera of 12 healthy donors (age 40–62 years; 75% female) was dried and redissolved in 10 µl of 100 mM Tris buffer (pH 8.5) containing 1% sodium deoxycholate, 10 mM Tris(2-carboxyethyl)phosphine, and 40 mM chloroacetamide. Denaturation, reduction, and alkylation was performed for 5 min at 95 °C. After cooling, 500 ng of sequencing grade trypsin (Promega) were added in 50 µl ammonium bicarbonate buffer (pH 8.5) and incubated overnight at 37 °C. Afterward, SDS was precipitated with 2% formic acid. Liquid chromatography–mass spectrometry (MS) analysis of IgA tryptic glycopeptides was performed as described previously^34^. In brief, (glyco-)peptides were separated on an Acclaim PepMap C18 column (particle size 2 μm, pore size 100 Å, 75 × 150 mm, Thermo Scientific) and analyzed on a quadrupole-time-of-flight-MS (Impact HD, Bruker Daltonics) coupled via a Captive Sprayer nano-electrospray ionization source (Bruker Daltonics). Data were processed with LaCyTools version 1.1.0-alpha, build 20181102b^58^. Healthy donor IgA glycopeptides were taken from previous reports, curated on basis of the existing data and relative quantities per glycosylation site were extracted^34^. Derived traits calculations can be found in the same report.

### Flow cytometry IgA binding studies to neutrophils and beads

Neutrophils were freshly isolated from EDTA-blood from healthy donors by standard density gradient centrifugation using Ficoll (Lymphoflot, BioRad) followed by two cycles of hypotonic erythrocyte lysis with sterile water. Purified neutrophils were kept in PBS supplemented with 2 mM EDTA and 2% FCS at a concentration of 2 × 10^6^ cells/ml and incubated with heat aggregated IgA at the indicated concentrations for 20 min at 4 °C. After washing, neutrophils were incubated with 1.25 µg/ml fluorescein isothiocyanate (FITC)-labeled goat F(ab)_2_ anti-human IgA (#2052-02; Southern Biotech) for 20 min at 4 °C.

Polystyrene beads (size 1 µm; Color Y; Estapor, Merck) were incubated with 100 µg/ml IgA in PBS for 1 h at room temperature and constant agitation. After washing, the beads were incubated with 1.25 µg/ml FITC-labeled goat F(ab)_2_ anti-human IgA (Southern Biotech) in PBS supplemented with 2 mM EDTA and 2% FCS for 20 min at 4 °C.

Flow cytometry was performed on a Gallios cytofluorometer and evaluated using Kaluza Analysis 2.1 software (both Beckman Coulter). Cell or bead aggregates, dead cells, and cell debris were excluded. Delta mean fluorescence intensity (ΔMFI) values were calculated by subtracting the MFI of neutrophils or beads stained with secondary antibody only.

### Enzymatic desialylation and deglycosylation of IgA

For desialylation, 1 mg of human IgA1 or IgA2 was incubated with 200 U α2-3,6,8 neuraminidase (NEB) for 18 h at 37 °C. For deglycosylation, 1 mg of human IgA1 or IgA2 was incubated with 1000 U PNGase F (NEB) for 18 h at 37 °C. The digestion efficiency was controlled with lectin blot analysis. Digested IgA was purified over a peptide M column (Invivogen) according to the manufacturer’s instructions and tested for endotoxin contamination using a LAL chromogenic endotoxin quantitation kit (Thermo scientific). Protein concentration was determined with the DC protein assay (Bio-Rad).

### Serum IgA quantification in healthy donors and patients with RA

Sera were obtained from patients with established RA presenting at the Department of Medicine 3 of the University Clinic of Erlangen and of age- and sex-matched healthy volunteers. Disease activity was evaluated using the DAS28 score. Concentrations of total serum IgA, IgA1, and IgA2 was determined by ELISA using goat F(ab)_2_ anti-human IgA (#2052-01) as capture and HRP-coupled goat anti-IgA (#2050-05), mouse anti-human IgA1 (#9130-05), or mouse anti-human IgA2 (#9140-05) as detection antibodies (all Southern Biotech). ACPA levels were determined using cyclic-citrullinated peptide-coated plates (Orgentec Diagnostika).

### Statistics

Statistical analysis was performed with GraphPad Prism 5.03 software. For comparison of two groups, two-sided Student’s *t*-test with Welch’s correction for unequal standard error or paired two-sided Student’s *t*-test was used. Statistics for three or more groups were calculated with one-way analysis of variance (ANOVA) followed by either Dunnet’s correction for all columns versus the control column or Bonferroni correction for selected pairs of columns. Correlations were investigated with Pearson’s correlation coefficient. *p* < 0.05 was considered significant. Data are presented as scatter plots with mean ± s.e.m. or box plots with medians and inter-quartile ranges + whiskers ranging from min to max. All analyses were performed in a blinded manner.

### Study approval

All analyses involving human blood samples were performed in accordance to the Declaration of Helsinki principles, the institutional guidelines and with the approval of the Ethical Committee of the University Clinic of Erlangen (Permit 277_17 B and 98_18 B). All individuals provided informed consent prior to participation in the study.

### Reporting summary

Further information on research design is available in the Nature Research Reporting Summary linked to this article.

## Supplementary information

Supplementary Information

Reporting Summary

## Data Availability

The authors declare that the data supporting the findings of this study are available within the paper and its supplementary information files or from the authors upon reasonable request. The raw data underlying all figures and tables are provided as a Source Data file.

## Source data

Source Data
