# Supplementary material for: IgA subclasses have different effector functions associated with distinct glycosylation profiles
Source: Nat Commun. 2020 Jan 8;11:120. doi: 10.1038/s41467-019-13992-8 (PMC6949214; doi:10.1038/s41467-019-13992-8)
Supplement: Supplementary file 1 — Supplementary Information [file 41467_2019_13992_MOESM1_ESM.pdf]

Supplementary Information for:

IgA subclasses display different effector functions associated with distinct glycosylation profiles

Steffen *et al*.

Contents:

page 2-9: Supplementary Figures 1-8

Page 10: Supplementary Table 1

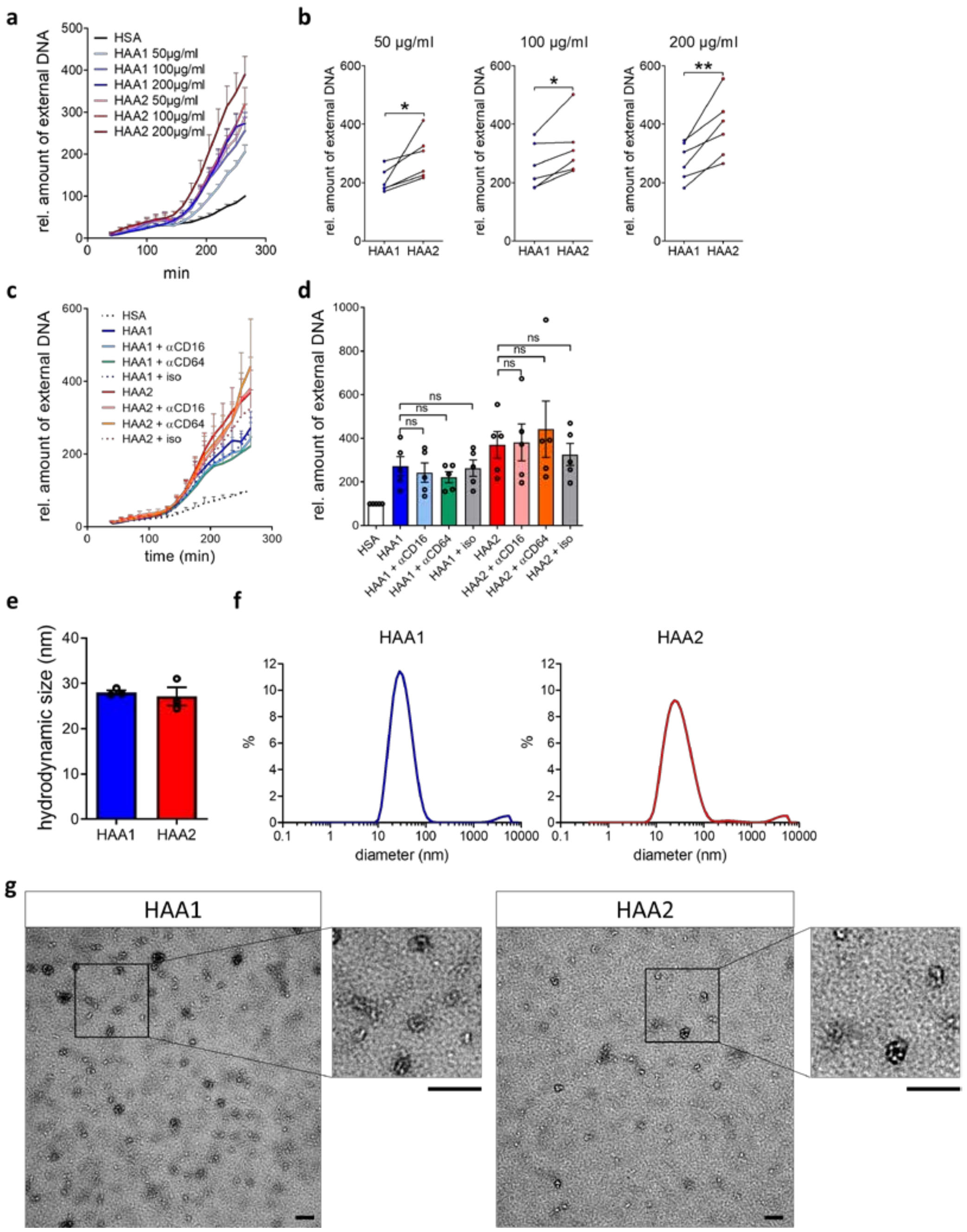

Supplementary Fig. 1: IgA complex mediated NET formation and characterization of IgA heat aggregates. (a+b) NET formation of human neutrophils with different concentrations of HAA1 or HAA2. (a) NET formation over time. (b) Relative amount of extracellular DNA at 265 min after stimulation normalized on HSA treatment. *n* = 6 donors. (c+d) NET formation of human neutrophils with 200 µg/ml HAA1 or HAA2 in the presence of 10 µg/ml blocking antibody against FcγRI (=αCD64), FcγRIII (=αCD16) or isotype control (=iso). (c) NET formation over time. (d) Relative amount of extracellular DNA at 265 min after stimulation normalized on HSA treatment. *n* = 5 donors. (e) Dynamic light scattering measurement of HAA1 and HAA2 size. *n* = 3 preparations. (f) Representative curves of the hydrodynamic diameter distribution of HAA1 and HAA2. (g) Representative electron microscopy images of HAA1 and HAA2. Scale bars = 100 nm. Significances were tested with paired two-sided Student's t test (b+e) or paired 1-Way ANOVA followed by Bonferroni correction for selected pairs of columns (d). * = p < 0,05; ** = p < 0,01; *** = p < 0,001. Data are presented as mean ± s.e.m. (a+c) or scatter plots with bars showing mean ± s.e.m (b,d+e). Source data are provided as a Source Data file.

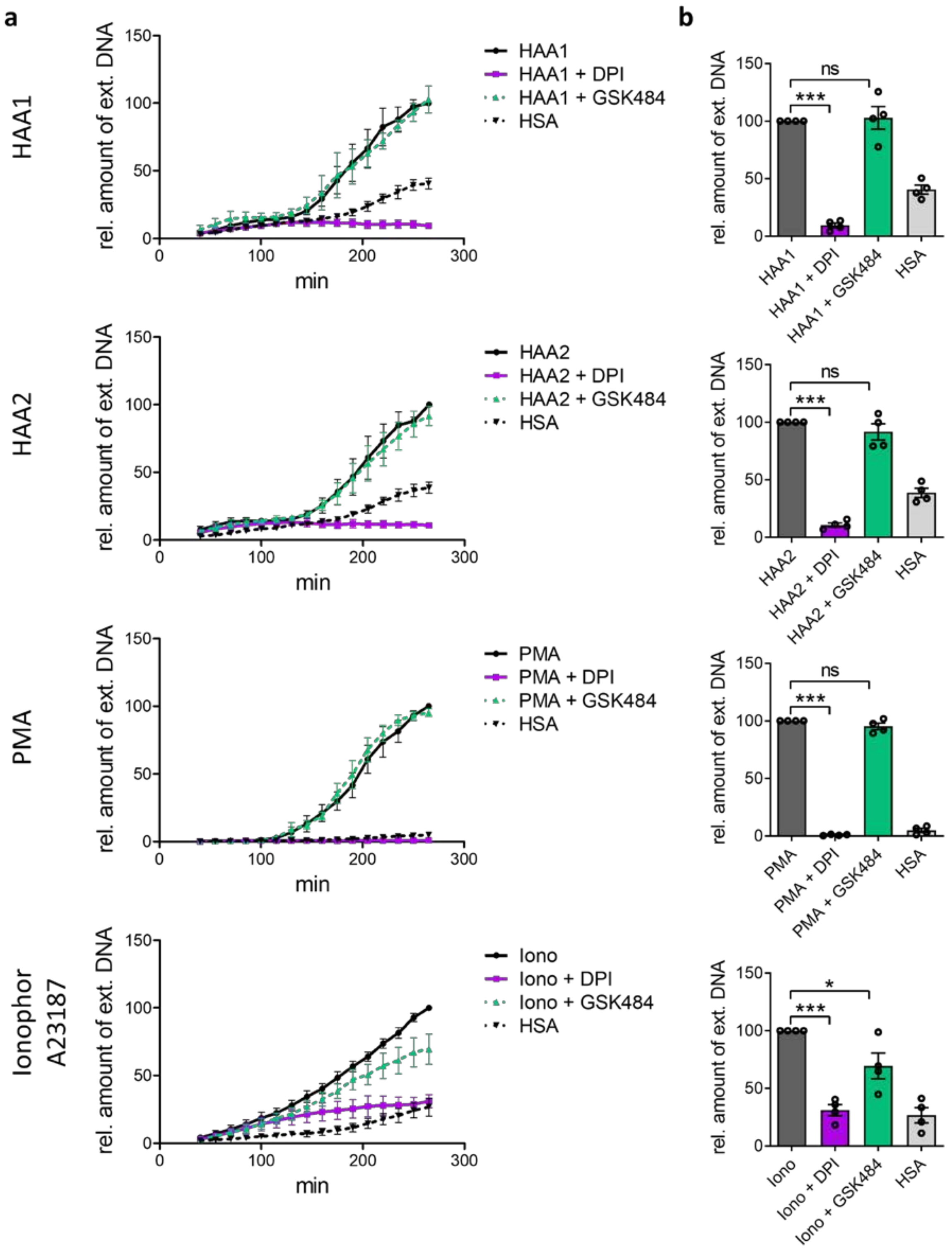

Supplementary Fig. 2: Characterization of pathways involved in IgA complex mediated NET formation. NET formation of human neutrophils with 200 µg/ml HAA1 or HAA2, PMA or ionophore A23187 (=Iono) in the presence of inhibitors for NOX (= DPI) or PAD4 (=GSK484). (a) NET formation over time. (b) Relative amount of extracellular DNA at 265 min after stimulation normalized on treatment without inhibitors. *n* = 4 donors. Significances were tested with paired 1-Way ANOVA followed by Bonferroni correction for selected pairs of columns. * = p < 0,05; ** = p < 0,01; *** = p < 0,001. Data are presented as mean ± s.e.m. (a) or scatter plots with bars showing mean ± s.e.m (b). Source data are provided as a Source Data file.

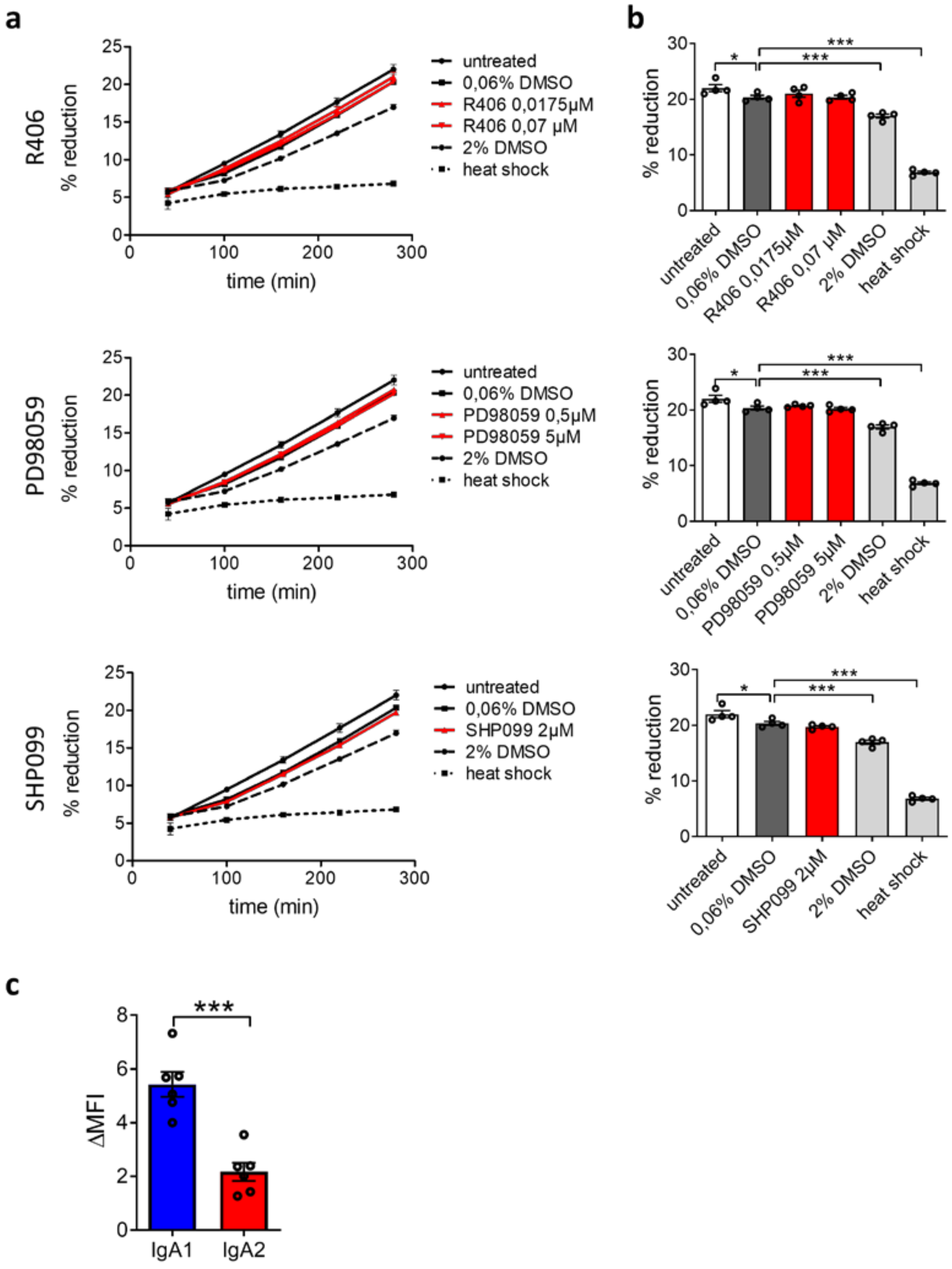

Supplementary Fig. 3: Viability test for kinase inhibitors and comparison of IgA1 and IgA2 recognition by the secondary antibody. (a+b) Viability test of neutrophils treated with inhibitors for Syk (= R406), Erk (= PD98059) or Shp-2 (= SHP099) or 0,06 % of DMSO as vehicle control in the presence of alamarBlue. As positive control, cells were treated with 2 % DMSO or pre-heated at 65°C. (a) Reduction of the absorbance of alamarBlue over time and (b) at 280 min. *n* = 4 donors c) Flow cytometry measurement of polystyrene beads coated with IgA1 or IgA2. *n* = 6 replicates from 2 independent experiments. Significances were tested with paired 1-Way ANOVA followed by Dunnett's correction using 0,06 % DMSO as control group (b) or two-sided Student's t test using Welch's correction (c). * = p < 0,05; ** = p < 0,01; *** = p < 0,001. Data are presented as mean ± s.e.m. (a) or scatter plots with bars showing mean ± s.e.m (b+c). Source data are provided as a Source Data file.

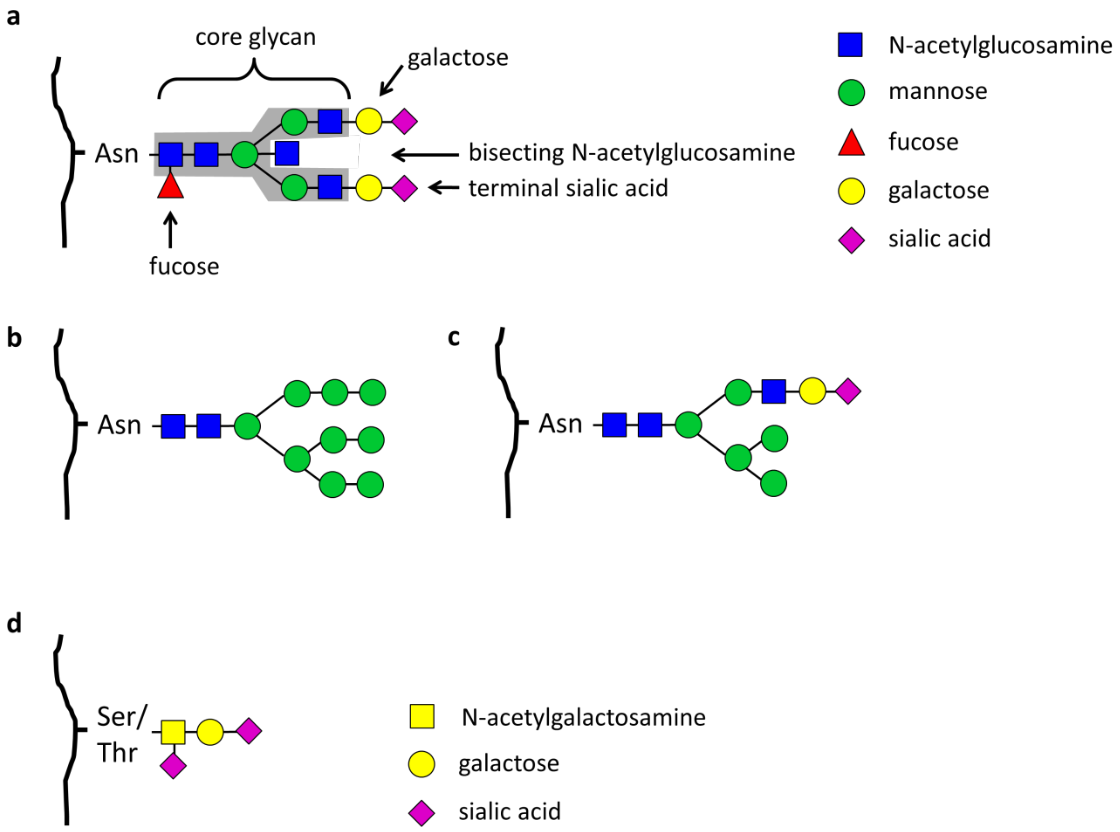

Supplementary Fig. 4: Schematic overview of glycan types. (a-c) Schematic overview of fully processed *N*-glycans of complex-type (a) high-mannose type (b) or hybrid-type (c). Complex type *N*-glycans typically contain a constant core domain (highlighted in grey) composed of 3 mannose and 4 N-acetylglucosamine residues to which fucose, a bisecting N-acetylglucosamine, galactose and sialic acid can be additionally attached. (d) Schematic overview of an *O*-glycan. Asn = aparagine; Ser = serine; Thr = threonine.

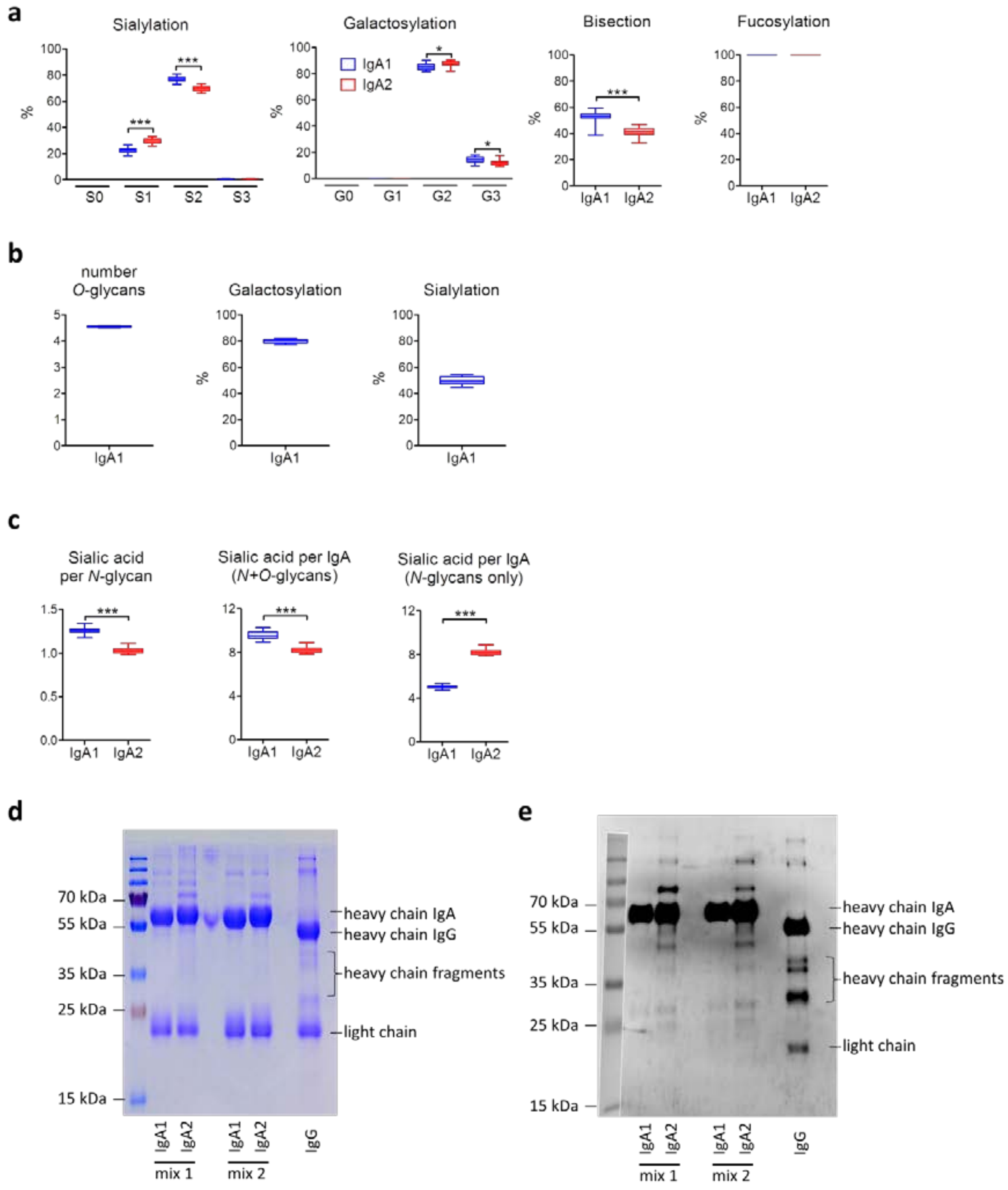

Supplementary Fig. 5: Additional glycosylation data. (a) Mass spectrometric quantification of sialyation, galactosylation, bisection and fucosylation from the truncated glycopeptide version of the glycosylation site N340/N327. (b) Mass spectrometric quantification of *O*-glycan numbers in IgA1 together with their galactosylation and sialylation. (c) Average amount of terminal sialic acid per *N*-glycan and the average number of sialic acid per IgA molecule regarding all glycans or only *N*-glycans. This calculation assumes complete occupation of the *N*-glycosylation sites. n = 12 donors. (d+e) Polyacrylamide gel electrophoresis of IgA1, IgA2 or IgG isolated from sera of healthy donors followed by staining with (d) Imperial™ Protein Stain or (e) lens culinaris agglutinin to detect protein and *N*-glycans, respectively. Significances were tested with paired 1-Way ANOVA followed by Bonferroni correction for selected pairs of columns (a) or paired two-sided Student's t test (a+b). * = p < 0,05; ** = p < 0,01; *** = p < 0,001. Data are presented as box plots with medians and inter-quartile ranges + whiskers ranging from min to max. Source data are provided as a Source Data file.

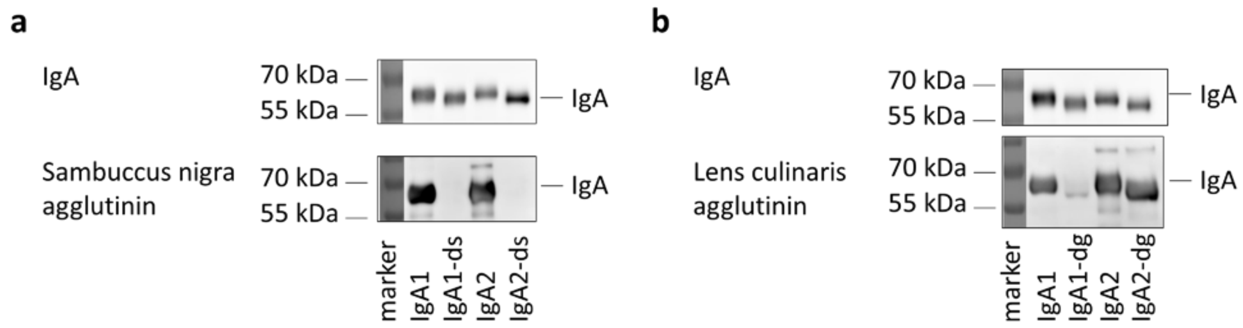

Supplementary Fig. 6: Verification of sugar removal from IgA. (a) Desialylation and (b) deglycosylation of IgA1 and IgA2 has been tested by lectin blotting with sambuccus nigra lectin detecting sialic acid and lens culinaris agglutinin detecting the core glycan. Staining against IgA was used as loading control. Source data are provided as a Source Data file.

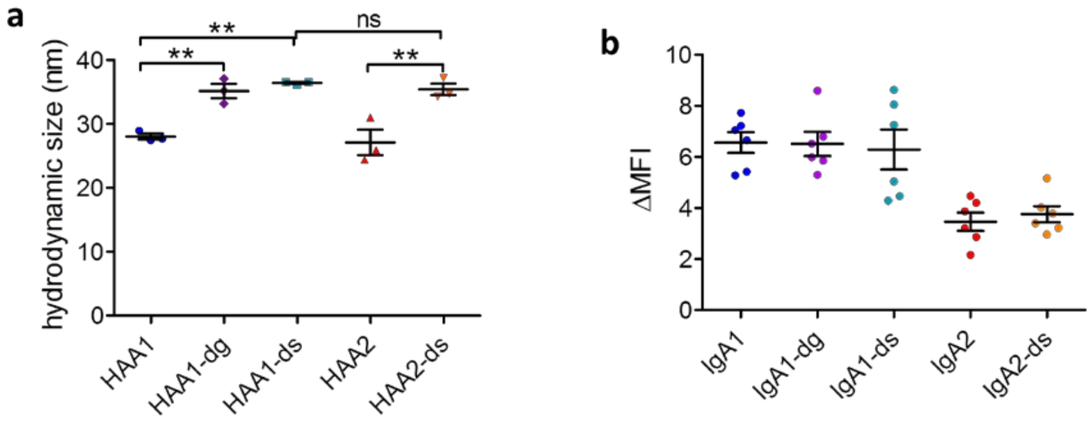

Supplementary Fig. 7: Verification of IgA1 and IgA2 size and recognition by the secondary antibody after sugar removal. (a) Dynamic light scattering measurement of HAA1 and HAA2 size before and after desialylation (=-ds) and deglycosylation (=-dg). *n* = 3 preparations. (b) Binding of IgA1 that has been desialylated or deglycosylated and IgA2 on polystyrene beads measured by flow cytometry. *n* = 6 replicates of 2 independent experiments. Significances were tested with paired 1-Way ANOVA followed by Bonferroni correction for selected pairs of columns. * = p < 0,05; ** = p < 0,01; *** = p < 0,001. Data are presented as scatter plots with mean ± s.e.m. Source data are provided as a Source Data file.

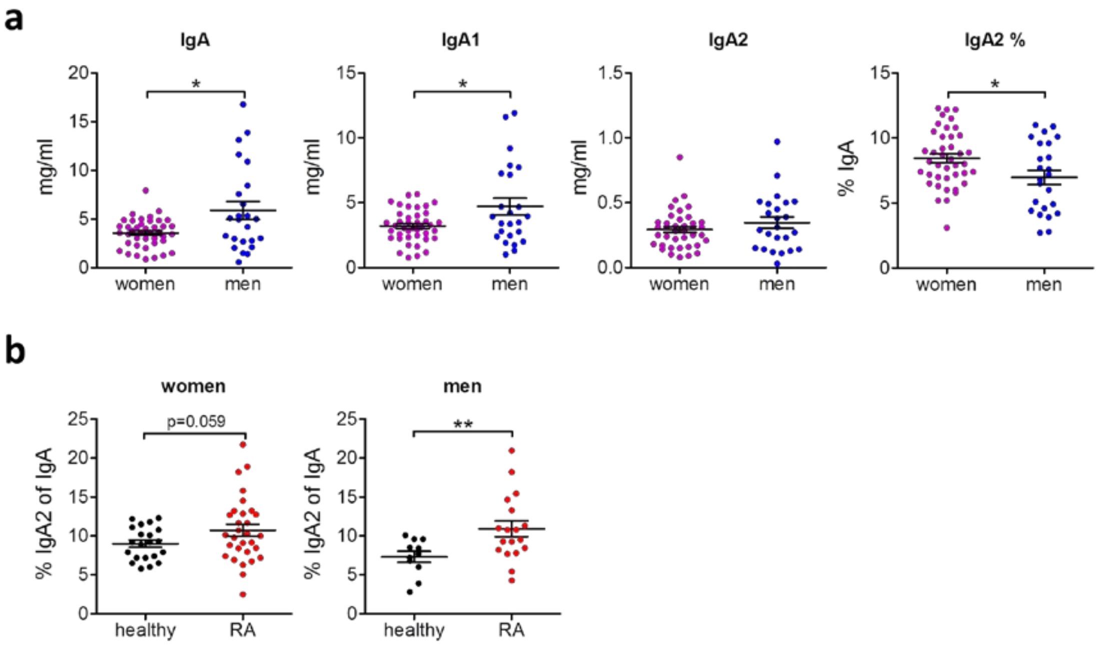

Supplementary Fig. 8: IgA subclass composition differs between men and women. (a) Amount of serum IgA, IgA1 and IgA2 as well as IgA2 percentage in healthy women (*n* = 41) and men (*n* = 24) of 20-80 years. (b) IgA2 percentage of total serum IgA in patients with rheumatoid arthritis (RA) and healthy controls grouped according to their sex. Healthy women: *n* = 21; RA women: *n* = 30; healthy men: *n* = 11; RA men: *n* = 18. Significances were tested with two-sided Student's t test using Welch's correction. * = p < 0,05; ** = p < 0,01; *** = p < 0,001. Data are presented as scatter plots with mean ± s.e.m. Source data are provided as a Source Data file.

Supplementary Table 1: Characteristics of healthy controls and patients with rheumatoid arthritis (RA).

	**Healthy Controls**	**All RA patients**	**ACPA+ RA patients**
**N**	32	48	38
**Age** (years)	54.5 (± 7.2)	55.7 (± 10.2)	55.6 (± 11.4)
**Sex** (females, %)	21 (65.6 %)	30 (62.5 %)	24 (63.2 %)
**DAS28** (units)	-	2.83 (± 1.17)	2.71 (± 1.06)

Values indicate mean ± SD. DAS28 = disease activity score 28; ACPA = anti-citrullinated protein antibodies. Source data are provided as a Source Data file.
