# Supplementary material for: IgA subclasses have different effector functions associated with distinct glycosylation profiles
Source: Nat Commun. 2020 Jan 8;11:120. doi: 10.1038/s41467-019-13992-8 (PMC6949214; doi:10.1038/s41467-019-13992-8)
Supplement: Supplementary file 2 — Reporting Summary [file 41467_2019_13992_MOESM2_ESM.pdf]

nature research

Corresponding author(s): Ulrike Steffen

Last updated by author(s): Nov 14, 2019

Reporting Summary

Nature Research wishes to improve the reproducibility of the work that we publish. This form provides structure for consistency and transparency in reporting. For further information on Nature Research policies, see Authors & Referees and the Editorial Policy Checklist .

Statistics

For all statistical analyses, confirm that the following items are present in the figure legend, table legend, main text, or Methods section.

n/a	Confirmed	
☐	☒	The exact sample size (*n*) for each experimental group/condition, given as a discrete number and unit of measurement
☐	☒	A statement on whether measurements were taken from distinct samples or whether the same sample was measured repeatedly
☐	☒	The statistical test(s) used AND whether they are one- or two-sided<br>*Only common tests should be described solely by name; describe more complex techniques in the Methods section.*
☒	☐	A description of all covariates tested
☐	☒	A description of any assumptions or corrections, such as tests of normality and adjustment for multiple comparisons
☐	☒	A full description of the statistical parameters including central tendency (e.g. means) or other basic estimates (e.g. regression coefficient) AND variation (e.g. standard deviation) or associated estimates of uncertainty (e.g. confidence intervals)
☐	☒	For null hypothesis testing, the test statistic (e.g. *F*, *t*, *r*) with confidence intervals, effect sizes, degrees of freedom and *P* value noted<br>*Give P values as exact values whenever suitable.*
☒	☐	For Bayesian analysis, information on the choice of priors and Markov chain Monte Carlo settings
☒	☐	For hierarchical and complex designs, identification of the appropriate level for tests and full reporting of outcomes
☒	☐	Estimates of effect sizes (e.g. Cohen's *d*, Pearson's *r*), indicating how they were calculated

Our web collection on statistics for biologists contains articles on many of the points above.

Software and code

Policy information about availability of computer code

Data collection	Adobe Photoshop CS5 extended (64 Bit)<br>Kaluza Analysis 2.1<br>LaCyTools version 1.1.0-alpha, build 20181102b
Data analysis	GraphPad Prism 5.03 and 8

For manuscripts utilizing custom algorithms or software that are central to the research but not yet described in published literature, software must be made available to editors/reviewers. We strongly encourage code deposition in a community repository (e.g. GitHub). See the Nature Research guidelines for submitting code & software for further information.

Data

Policy information about availability of data

All manuscripts must include a data availability statement. This statement should provide the following information, where applicable:
- Accession codes, unique identifiers, or web links for publicly available datasets
- A list of figures that have associated raw data
- A description of any restrictions on data availability

All data is available from the authors upon reasonable request. The source data underlying all Figures, Supplementary Figures and Supplementary Tables are provided as a Source Data file.

Field-specific reporting

Please select the one below that is the best fit for your research. If you are not sure, read the appropriate sections before making your selection.

Life sciences study design

All studies must disclose on these points even when the disclosure is negative.

Sample size	Sample size was determined based on empirical values from previous studies.
Data exclusions	No data were excluded.
Replication	We have performed the experiments with different donors to prove reproducibility. Although the effector size was dependent on the donor, experimental outcomes were reproducible.
Randomization	We did not need random allocation, as cells from the same donor were stimulated with different reagents. Therefore, each experimental setup had internal controls.<br>In case patients were compared with healthy controls, samples from both groups were evenly distributed on the assay plate setup.
Blinding	Data were analysed in a blinded manner.

Reporting for specific materials, systems and methods

We require information from authors about some types of materials, experimental systems and methods used in many studies. Here, indicate whether each material, system or method listed is relevant to your study. If you are not sure if a list item applies to your research, read the appropriate section before selecting a response.

Materials & experimental systems

n/a	Involved in the study
☐	☒ Antibodies
☒	☐ Eukaryotic cell lines
☒	☐ Palaeontology
☒	☐ Animals and other organisms
☐	☒ Human research participants
☒	☐ Clinical data

Methods

n/a	Involved in the study
☒	☐ ChIP-seq
☐	☒ Flow cytometry
☒	☐ MRI-based neuroimaging

Antibodies

Antibodies used

- human serum IgA1; in house isolated from human serum
- human serum IgA2; in house isolated from human serum
- Mouse anti Human CD89 - Low Endotoxin; clone MIP8a; BioRad; #MCA1824EL; LOT 1709
- Mouse anti-human CD16 - LEAF; clone 3G8; Biolegend; #302013; LOT B269541
- Mouse anti-human CD64 - LEAF; clone 10.1; Biolegend; #305016; LOT B131618
- Mouse IgG1, κ Isotype Ctrl Antibody - LEAF; clone MG1-45; Biolegend; #401404; LOT B124396
- Goat F(ab')2 Anti-Human IgA; Southern Biotech; #2052-01; LOT G2713-VB37
- Goat F(ab')2 Anti-Human IgA-FITC; Southern Biotech; #2052-02; LOT G2713-WS756
- Goat Anti-Human IgA-HRP; Southern Biotech; #2050-05; LOT C5213-Q395F
- Mouse Anti-Human IgA1-HRP; Southern Biotech; #9130-05; LOT F3017-VA77C
- Mouse Anti-Human IgA2-HRP; Southern Biotech; #9140-05; LOT J2113-NE15C
- Biotinylated Lens Culinaris Agglutinin (LCA); Vector Laboratories; #B-1045; LOT ZC1221
- Biotinylated Sambucus Nigra Agglutinin (SNA, EBL); Vector Laboratories; #B-1305; LOT X0220
- Biotinylated Erythrina Cristagalli Lectin (ECL, ECA); Vector Laboratories; #B-1145; LOT ZB0420

Validation

- human serum IgA1 and IgA2: purity was measured with western blot analysis using antibodies against IgA1 and IgA2 as well as coomassie staining to detect contaminations of foreign proteins. LPS was depleted using Triton X-114 and Triton was removed with detergent removal columns.
- Mouse anti Human CD89 - Low Endotoxin: see https://www.bio-rad-antibodies.com/monoclonal/human-cd89-antibody-mip8a-mca1824.html?f=low%20endotoxin
- Mouse anti-human CD16 - LEAF: see https://www.biolegend.com/nl-nl/products/leaf-low-endotoxin--azide-freepurified-anti-human-cd16-antibody-568
- Mouse anti-human CD64 - LEAF: see https://www.biolegend.com/de-de/products/ultra-leaf-purified-anti-human-cd64-antibody-17729
- Mouse IgG1, κ Isotype Ctrl Antibody - LEAF: see https://www.biolegend.com/en-us/products/ultra-leaf-purified-mouse-igg1--kappa-isotype-ctrl-7729
- Goat F(ab')2 Anti-Human IgA: see https://www.southernbiotech.com/?catno=2052-01&type=Polyclonal#&panel2-1
- Goat F(ab')2 Anti-Human IgA-FITC: see https://www.southernbiotech.com/?catno=2052-02&type=Polyclonal#&panel2-1
- Goat Anti-Human IgA-HRP; Southern Biotech: see https://www.southernbiotech.com/?

catno=2050-05&type=Polyclonal#&panel2-1
- Mouse Anti-Human IgA1-HRP: see https://www.southernbiotech.com/?catno=9130-05&type=Monoclonal#&panel2-1
- Mouse Anti-Human IgA2-HRP: see https://www.southernbiotech.com/?catno=9140-05&type=Monoclonal#&panel2-1
- Biotinylated Lens Culinaris Agglutinin (LCA): see https://vectorlabs.com/biotinylated-lens-culinaris-agglutinin-lca.html
- Biotinylated Sambucus Nigra Agglutinin (SNA, EBL): see https://vectorlabs.com/biotinylated-sambucus-nigra-lectin-sna-ebl.html
- Biotinylated Erythrina Cristagalli Lectin (ECL, ECA): see https://vectorlabs.com/biotinylated-erythrina-cristagalli-lectin-ecl-eca.html

Human research participants

Policy information about studies involving human research participants

Population characteristics	For human studies, patients with established RA presenting at the Department of Medicine 3 of the University Clinic of Erlangen and age- and sex-matched healthy volunteers were involved. Mean age was about 55 years and females made about 63% of the population.<br>For experiments with primary human cells, blood was taken from healthy volunteers (male and female) at ages between 20 and 50 years.
Recruitment	Patients with established RA were randomly choosen from the serum bank of the Department of Medicine 3 of the University Clinic of Erlangen.<br>Healthy controls and volunteers were recruited in Erlangen using flyers.<br>All subjects provided informed consent prior to their participation in the study.
Ethics oversight	The studies were approved by the Ethical Committee of the University Clinic of Erlangen.

Note that full information on the approval of the study protocol must also be provided in the manuscript.

Flow Cytometry

Plots

Confirm that:

- [x] The axis labels state the marker and fluorochrome used (e.g. CD4-FITC).
- [x] The axis scales are clearly visible. Include numbers along axes only for bottom left plot of group (a 'group' is an analysis of identical markers).
- [x] All plots are contour plots with outliers or pseudocolor plots.
- [x] A numerical value for number of cells or percentage (with statistics) is provided.

Methodology

Sample preparation	Neutrophils were freshly isolated from EDTA-blood from healthy donors by standard density gradient centrifugation using Ficoll (Lymphoflot, BioRad) followed by 2 cycles of hypotonic erythrocyte lysis with sterile water. Purified neutrophils were kept in PBS supplemented with 2 mM EDTA and 2 % FCS at a concentration of 2 x 106 cells per ml and incubated with heat aggregated IgA at the indicated concentrations for 20 min at 4°C. After washing, neutrophils were incubated with 1.25 µg/ml fluorescein (FITC)-labeled goat F(ab)2 anti-human IgA (#2052-02; Southern Biotech) for 20 min at 4°C.
Instrument	Flow cytometry was performed on a Gallios cytofluorometer (Beckmann Coulter)
Software	Kaluza Analysis 2.1
Cell population abundance	Purity of polymorph-nuclear cells after isolation was >95% (determined by cell counting and flow cytometry analsys.
Gating strategy	FSC/SSC plots were used to exclude cell debris defined as very small events.

- [x] Tick this box to confirm that a figure exemplifying the gating strategy is provided in the Supplementary Information.
